# Supplementary material for: IP3 mediated global Ca2+ signals arise through two temporally and spatially distinct modes of Ca2+ release
Source: eLife. 2020 May 12;9:e55008. doi: 10.7554/eLife.55008 (PMC7253181; doi:10.7554/eLife.55008)
Supplement: Supplementary file 1. [file elife-55008-supp1.docx]

**SUPPLEMENTARY FILE 1**

**Ca^2+^ Image Processing Routines**

The following texts are executed as scripts within the FLIKA software package (<https://github.com/flika-org/flika>) to generate image stacks representing temporal and spatial fluctuations in Ca^2+^ image data.

**Temporal SD fluctuations algorithm**

#parameters#

sigma = 2 **#** *In pixels.* *Change this number to vary the strength of the gaussian blur*

sampling_interval = 10 **#** *frame duration in ms*

q = 0.186 **#** *scalar constant to correct for photon-shot noise, derived as described in Fig. S1*

low_cutoff = 3 **#** *low cut off for Butterworth filter in Hz*

high_cutoff = 20 **#** *high cut off Butterworth filter in Hz*

filter_order = 3 **#** *increasing filter order increases the steepness of the Butterworth filter*

boxcar_width = 160 **#** *in milliseconds*

#red text denotes user specified parameters#

# run after specifying above parameters and subtracting black level #

from scipy.ndimage.filters import convolve

sampling_rate = 1/(sampling_interval/1000) # in Hz

try:

assert high_cutoff <= .5 * sampling_rate

except AssertionError:

print('High Frequency Cutoff is above the Nyquist frequency. Lower your high frequency cutoff')

high_cutoff_scaled = high_cutoff / (sampling_rate/2)

low_cutoff_scaled = low_cutoff / (sampling_rate/2)

boxcar_frames = int(np.round(boxcar_width / sampling_interval))

#A = np.sqrt(10) * np.random.randn(10000, 10,10) + 10

#Window(A, 'original image')

nFrames = g.win.mt

prefilter = gaussian_blur(sigma, keepSourceWindow=True)

postfilter = butterworth_filter(filter_order, low_cutoff_scaled, high_cutoff_scaled, keepSourceWindow=True)

A = prefilter.image

B = postfilter.image

mean_A = convolve(A, weights=np.full((boxcar_frames,1,1),1.0/boxcar_frames))

mean_B = convolve(B, weights=np.full((boxcar_frames,1,1),1.0/boxcar_frames))

variance_B = np.zeros_like(B)

print('Calculating variance')

for i in np.arange(nFrames):

print('{}/{}'.format(i, nFrames))

mean_frame = mean_B[i]

i0 = int(i-boxcar_frames/2)

if i0<0:

i0 = 0

i1 = int(i+boxcar_frames/2)

if i1 > nFrames:

i1 = nFrames

cutout_frames = B[i0:i1]

variance_B[i] = np.mean((cutout_frames - mean_frame)**2, 0)

sqrt_B = np.sqrt(variance_B)

mean_A[mean_A<0] = 0 #removes negative values

sqrt_Bf = sqrt_B - np.sqrt(q*mean_A)

Window(sqrt_Bf, 'stdev minus sqrt mean')

#Window(variance_B - (q*mean_A), 'Variance minus mean')

**Spatial SD fluctuations algorithm**

subtract(500, keepSourceWindow=False) **#** *camera black level*

trim(1000, 4000, increment=1, delete=False, keepSourceWindow=False) **#** *removes frames from beginning and ending of image stack*

I1=trim(1327, 1345, increment=1, delete=True, keepSourceWindow=False) **#** *removes flash artifact*

multiply(0.11, keepSourceWindow=True) **#** *scaled raw fluorescence*

I2=sqrt(2)

I1.setAsCurrentWindow()

butterworth_filter(3, 3, 20, 125, keepSourceWindow=True)

I3=gaussian_blur(2, 8, keepSourceWindow=False) # *sigma values used to calculate the difference of Gaussian blur functions*

image_calculator(I3,I3,'Multiply',keepSourceWindow=True)

I4=sqrt(2)

image_calculator(I4,I2,'Subtract',keepSourceWindow=True)

#red text denotes user defined parameters#
